# Maraviroc/cisplatin combination inhibits gastric cancer tumoroid growth and improves mice survival

**DOI:** 10.1186/s40659-024-00581-3

**Published:** 2025-01-18

**Authors:** Bárbara Mora-Lagos, María Elena Reyes, Lorena Lobos-Gonzalez, Matías del Campo, Kurt Buchegger, Louise Zanella, Ismael Riquelme, Carmen Gloria Ili, Priscilla Brebi

**Affiliations:** 1 Instituto de Ciencias Biomédicas, Facultad de Ciencias de la Salud, Universidad Autónoma de Chile, Temuco, Chile; 2 Centro de Medicina Regenerativa, Facultad de Medicina-Clínica Alemana, Universidad del Desarrollo, Santiago, Chile; 3 Advanced Center for Chronic Diseases, ACCDiS, Santiago, Chile; 4 Laboratorio de comunicaciones celulares, Instituto de Ciencias Biomédicas, iCBM, Universidad de Chile, Santiago, Chile; 5 Department of Basic Sciences, Faculty of Medicine, Universidad de La Frontera, Temuco, Chile; 6 Laboratory of Integrative Biology (LIBi), Centro de Excelencia en Medicina Traslacional (CEMT), Scientific and Technological Bioresource Nucleus (BIOREN), Universidad de La Frontera, Temuco, Chile; 7 Millennium Institute on Immunology and Immunotherapy, Santiago, Chile; 8 Doctorado en Ciencias Médicas, Universidad de La Frontera, Temuco, Chile; 9 Núcleo Milenio de Sociomedicina, Santiago, Chile; 10 Biomedical Research Consortium-Chile (BMRC), Santiago, Chile

**Keywords:** Gastric cancer (GC), Cisplatin (CDDP), Chemoresistance, C-C chemokine receptor type 5 (CCR5), C-C motif chemokine ligand 5 (CCL5), Maraviroc (MVC)

## Abstract

**Background:**

Gastric cancer (GC) is a significant cancer-related cause of death worldwide. GC’s most used chemotherapeutic regimen is based on platinum drugs such as cisplatin (CDDP). However, CDDP chemoresistance reduces the survival rate of advanced GC. The immune C-C chemokine receptor type 5 (CCR5) have been proposed as a pivotal factor in cancer progression since its blockade has been linked with antineoplastic effects on tumor cell proliferation; nevertheless, its role in the chemoresistance of GC has not been elucidated. This study aimed to determine the effects induced by the CCR5 using Maraviroc (MVC), a highly selective CCR5 antagonist, on CDDP-resistant AGS cells (AGS R-CDDP), tumoroids (*3D* tumor spheroids), and animal models.

**Results:**

The combined CDDP and MVC treatment reduced cell viability and inhibited tumoroid formation in AGS R-CDDP cells. The effects of the MVC/CDDP combination on apoptosis and cell cycle progression were correlated with the increase in CDDP (dose-dependent). The mRNA levels of C-C Motif Chemokine Ligand 5 (CCL5), the main ligand for CCR5, decreased significantly in cells treated with the MVC/CDDP combination. MVC in the MVC/CDDP combination improved the survival rate and biochemical parameters of CDDP-treated mice by reducing the side effects of CDDP alone.

**Conclusions:**

This finding suggests that MVC/CDDP combination could be a potential complementary therapy for GC.

**Supplementary Information:**

The online version contains supplementary material available at 10.1186/s40659-024-00581-3.

## Background

Gastric cancer (GC) is the fifth most common cancer in terms of incidence and the fourth most common cancer in terms of mortality, representing a serious public health concern in the world [1]. Surgery is an effective treatment for the early stages of GC, notwithstanding chemotherapy is the central strategy for patients diagnosed in advanced or metastatic stages to improve survival rates and mitigate adverse symptoms [2, 3]. Cisplatin (CDDP) is a widely used drug in first-line therapy against advanced GC [4, 5]. This platinum compound binds covalently to DNA, forming adducts that inhibit DNA replication, suppress transcription, induce cell-cycle arrest, disrupt DNA damage repair, and promote apoptosis [6]. Unfortunately, chemotherapy has shown limited benefits in advanced stage GC, reaching an average survival of ten months, due to the reduced treatment efficiency, which can lead to tumor regrowth and lower patient survival [7]. Chemoresistance represents the most important cause of cancer treatment failure and mortality [8]. The principal mechanisms that contribute to developing CDDP chemoresistance include: (i) increase the DNA repair, (ii) increase CDDP efflux rate, (iii) decrease the expression of the principal CDDP influx transporter (CTR1), (iv) CDDP detoxication by metallothioneins and glutathione molecules, (v) inactivated cell death signaling [9]; and (vi) epigenetic regulation [10]. In the effort to identify new mechanisms of CDDP resistance and find potential therapeutic targets, the role of the CC chemokine subfamily in CDDP chemoresistance has been investigated [11]. Previous studies, performed by our group, established, and characterized a new gastric cancer cell line resistant to CDDP through functional assays and RNA-seq analysis. These studies allowed us to elucidate the relationship between CDDP chemoresistance and the immune system. We found that the majority of differentially expressed genes (DEGs), such as C-C Motif Chemokine Ligand 5 (CCL5), were enriched in the inflammation mediated by chemokine and cytokine signaling pathway. Moreover, we found that CCL5 mRNA expression was upregulated in these chemoresistant gastric cancer cells [12]. CCL5, also known as Regulated upon Activation, Normal T-cell Expressed and Secreted (RANTES), is a chemokine secreted in a paracrine or autocrine fashion that plays an active role in recruiting a variety of leukocytes into inflammatory sites [13]. CCL5 and its receptor CCR5 (C-C chemokine receptor type 5) constitute an axis (CCL5/CCR5) whose role has been extensively studied in cancer progression. In this regard, this manuscript focused on the CCL5 receptor (CCR5), a G protein-coupled receptor that mediates the physiological functions of immune cells. CCR5 has been involved in the stimulation of proliferation, invasion, and metastasis in GC [14], and its overexpression has been observed in many types of cancers [13, 15], including GC [16]. CCR5 is a co-receptor of Human immunodeficiency virus (HIV-1) in immune cells and therapies targeting CCR5 include Maraviroc (MVC), a small molecule FDA-cleared for the treatment of patients with HIV [13, 15]. In cancer, CCR5 has been implicated in hematological cancers [17] and solid tumors, mainly breast cancer [18], colorectal cancer [19], and GC [20]. In the case of colorectal cancer, studies using the SW480 and SW620 cell lines demonstrated that CCR5 blockade with MVC reduced cell viability. Additionally, Pervaitz et al.. showed that CCR5 antagonism by MVC induces significant apoptotic effects in colorectal cancer cells [21]. Regarding gastric cancer, this receptor has been linked to the progression and dissemination of this type of cancer. In a study using an in vitro model with AGS cells to investigate the modulation of the CCL5/CCR5 axis, it was found that receptor expression increased with rising chemokine levels in the tumor microenvironment [22]. CCR5 blockade by MVC has been studied in GC progression in an in vivo model, with effects such as reduced the number and total volume of peritoneal and mesenteric nodules, increased median survival time, and induced extensive intratumoral necrosis in mice [16, 23], however, it has not been fully elucidated in chemoresistance models. Moreover, we investigated the effects generated by the blockade of CCR5 by MVC by itself and in combination with CDDP, using different models of chemoresistance in GC, including CDDP-resistant AGS cells (AGS R-CDDP), tumoroids (3D tumor spheroids), and animal model. Our results showed that the use of the MVC/CDDP combination resulted in an improved survival rate and more favorable biochemical parameters in mice undergoing CDDP treatment. This outcome suggests that the MVC/CDDP combination could represent a promising complementary therapy for GC, given its ability to reduce CDDP side effects.

## Materials and methods

### Drugs

Cisplatin (CDDP; Cat# 15663-27-1) and Maraviroc (MVC; Cat# 376348-65-1) were purchased from Sigma Aldrich (Merck group, Germany). CDDP was reconstituted at a concentration of 3.3 mM diluted in 0.9% (p/v) NaCl, and aliquots of stock solution were stored at -80 °C. We used a high (26.05 µM) and a low (4.8 µM) concentration of CDDP, corresponding to the EC_50_ of AGS R-CDDP cells and AGS WT cells, respectively, achieved after 72 h of incubation [12]. MVC was reconstituted at a concentration of 5µM following Mercanelli et al. instructions [23].

### Cell lines culture

We utilized two gastric cancer cell lines in this study: AGS WT (wild type), and AGS CDDP-resistant. AGS WT, a human Caucasian Gastric Adenocarcinoma cell line, Cat# 8,909,040, was acquired by the European Collection of Authenticated Cell Cultures (ECACC) (distributed by Sigma-Aldrich Corporation-ECACC, Oceania Inventory). AGS R-CDDP cell line was established from the AGS WT cell line by increasing CDDP drug doses stepwise and posterior characterization by functional and transcriptomic analyses [12]. AGS WT and AGS R-CDDP were cultured in RPMI-1640 medium (Cat# 22400071, ThermoFisher, USA) supplemented with 10% (v/v) fetal bovine serum (Cat# 16000044, ThermoFisher, USA) and 1% (v/v) penicillin and streptomycin (Cat# 15140122, ThermoFisher, USA). Cells were maintained at 37 °C in a 95% humidified atmosphere and 5% CO_2_ conditions. Both resistant and wild-type cell lines were subcultured at 80% confluence and harvested after treatment with 0.25% trypsin and 0.02% EDTA (Cat# 25200056, ThermoFisher, USA).

### Viability assays

Viability assays were performed using a standard viability assay (MTT assay). Briefly, 5.5 × 10^3^ cells were seeded in 96-well plates in 100 µL of culture medium and incubated for 24 h to allow cell attachment until reaching 50% confluence. The cells were exposed 24, 48, and 72 h to MVC (5µM) and/or CDDP (26.05 µM and 4.8 µM). Cells incubated with 0.9% (p/v) NaCl were used as controls. After each incubation, the culture medium was removed, and the cells were washed with 100 µL of DPBS/Modified (Cat. 21-031-CM, Corning, USA) and subsequently treated with MTT (Cat. 298-93-1, Merck group, Germany) at 0.5 mg/mL, followed by 2 h of incubation at 37° C. Only functional mitochondrial dehydrogenase enzymes from viable cells can reduce MTT to form formazan, evidenced by the formation of a purple precipitate, and 100 µL of propanol is used to dissolve it entirely. Absorbance was measured at 570 nm wavelength using the Infinite NanoQuant spectrophotometer (TECAN, Switzerland).

### Cell death assay

Cell death assay was performed using the Dead Cell Apoptosis Kit with Alexa Fluor™ 488 annexin V and propidium iodide (PI) (Cat. V13245, Invitrogen, USA) according to the manufacturer’s instructions. Briefly, 7 × 10^4^ cells were seeded in 6-well plates in 2 mL of culture medium and incubated for 24 h to allow cell attachment. Then, cells were exposed 72 h to MVC (5µM) and/or CDDP (26.05 µM and 4.8 µM). Cells with 0.9% (p/v) NaCl were used as controls. Following incubation, cells were harvested by trypsin treatment and centrifuged. The pellet obtained was washed with 1X DPBS (Cat# 21-031-CM, Corning, USA) and resuspended in 100 µL of 1X annexin buffer. For staining, cells were incubated with 5 µL of Alexa Fluor^®^ 488 annexin V and 1 µL of 100 µg/mL Propidium Iodine (PI) at 37˚C for 15 min. Finally, cells were resuspended in 400 µL of 1X annexin buffer and collected for analysis by flow cytometry (FACSCANTO II, BD, USA). As apoptosis-inducing agent, 5% DMSO (Cat. DI-0755, Winckler, Chile) was used. Early apoptotic cells (annexin V-positive, PI-negative), late apoptotic cells (annexin V-positive and PI-positive), annexin V-negative and PI-positive cell populations were all considered dead cells. The fluorescence was read at maximum excitation/emission of 499⁄521 for Alexa Fluor^®^ 488 annexin V and 535⁄617 for PI.

### Cell cycle assay

Cell cycle assay was performed using Muse^®^ Cell Cycle Assay Kit (Cat. CB. MCH100106, Merck Millipore, USA), according to the manufacturer’s protocol. The kit utilizes propidium iodide (PI) staining to allow quantitative measurements of the percentage of cells in the G0/G1, S, and G2/M phases. Briefly, 7 × 10^4^ resistant cells were seeded in 6-well plates in 2 mL of culture medium and incubated for 24 h to allow cell attachment. Cells were synchronized through serum starvation for 19 h. Then, cells were exposed 24, 48, and 72 h to MVC (5µM) and/or CDDP (26.05 µM and 4.8 µM). Cells incubated with 0.9% (p/v) NaCl were used as controls. Following incubation, cells were harvested by trypsin treatment and centrifuged. The pellet obtained was washed with 1X DPBS (Cat# 21-031-CM, Corning, USA), resuspended in 1mL of cold 70% ethanol, and stored at -20 °C. For staining, 200 µL of fixed cells were taken, centrifuged, and washed twice with DPBS. The pellet was resuspended in 200 µL of Muse^®^ Cell Cycle reagent (premixed reagent that includes PI staining for DNA and RNase A intercalation) and incubated for 30 min at room temperature, protected from light. The samples were analyzed by flow cytometry in a Muse™ Cell Analyzer (EMD Millipore Bioscience, USA).

### RNA extraction and quantitative analysis

The mRNA expression of *CCL5* was quantified by RT-qPCR. Pellet was collected 72 h after treatments with MVC (5µM) and/or CDDP (26.05 µM and 4.8 µM). The selection of this time point is due to the fact that the most significant decreases in cell viability with the combined treatment of MVC and CDDP were observed at 72 h. Cells incubated with 0.9% (p/v) NaCl were used as control. Total RNA was extracted from ~ 2.0 × 10^6^ cells using TRIzol Reagent (Cat# 15596018, ThermoFisher, USA) according to the manufacturer’s instructions. RNA concentration was quantified using the Infinite^®^NanoQuant spectrophotometer (TECAN, Switzerland), and integrity was evaluated by measuring RNA 260/280 absorbance ratio and gel electrophoresis. Then, RNA was treated with DNase I (Cat.M6101, Promega Corp, USA), and the first-strand cDNA was prepared from 1 µg of RNA in a total reaction volume of 20 µL using M-MLV reverse transcriptase 200 U/µL (Cat. M1701, Promega Corp, USA) at 42 ºC for 60 min. Subsequently, cDNA was amplified by qPCR using Brilliant II Ultra-Fast SYBR^®^ Green qPCR Master Mix (Cat# 600828, Agilent Technologies, USA) according to the manufacturer’s protocol, using the Stratagene Mx-3000p real-time PCR system (Agilent Technologies, USA). Relative expression was determined by 2^−ΔΔCT^ method, using *ACTB* as the reference gene. Sequences of oligonucleotides used in this study are detailed in Supplementary Table 1.

### Tumoroid formation assay

Tumoroids (*3D* tumor spheroids) were grown from AGS R-CDDP cells seeded in low adhesion plates (Nunclon™ Sphera™, Thermofisher, USA) supplemented with Mammary Epithelial Cell Growth culture medium (at least 1.5 mL of MEGM™, Cat. CC-3151, Lonza, Switzerland), EGF 25 ng/mL, hydrocortisone 0.5 g/mL, insulin 5 µg/mL (Cat. CC-4136, Lonza, Switzerland) and bFGF 25 ng /mL (Cat. PHG0026, Invitrogen, USA). For tumoroid expansion, AGS R-CDDP were seeded on sterile 2% agar-covered plates (6 well-plates). Tumoroids were grown at 37 °C and 5% CO_2_ for 14 days. The inclusion/exclusion criteria are based on the diameter of the tumoroid: by day 7, the tumoroid should measure over 50 μm, and by day 14, over 100 μm as described by Durán-Jara et al. [24]. Cell culture medium was not renewed during the 14 days of the experiment, and the formation of spheres was visually and recorded by photography using the Micrometrics SE Premium 4 software (Accu-Scope, USA) in a Nikon Eclipse TS100 inverted microscope (Nikon Instruments Inc., USA). After 14 days, cell culture medium with the spheres was extracted from wells and passed through a 70 μm filter (BD Falcon, USA). Pharmacological stimuli with MVC and/or CDDP were added on days 14, 17, and 20. The final count of tumoroids was performed on day 24, and the formation of spheres was observed. We used a high and low concentration for each drug (MVC: 10 µM and 5 µM, CDDP: 26.05 µM and 4.8 µM). Cells not treated (NT) and cells exposed to DMSO were used as controls. The tumoroid standardization assay focused on determining the tumoroid formation capacity in AGS R-CDDP compared to AGS WT cells is shown in Supplementary Fig. 1.

### Animal model

Animal studies were conducted in accordance with the Ethical Committee of the Universidad Del Desarrollo (Approval certificate Nº07/2021_CICUAL-UDD). Immune-compromised BalbC NOD/SCID mice (males and females) were obtained from The Jackson Laboratory (Bar Harbor, ME, USA) and maintained in the animal facilities of the Universidad Del Desarrollo under specific pathogen-free conditions, in a temperature-controlled environment with 12/12 h light/dark schedule. Animals were fed with sterile food and water *ad libitum.*

### Tumor growth assay

Mice between 6 and 8 weeks of age were used for the experiments. AGS R-CDDP cells were cultured to develop tumoroids at 14th days. The tumoroids were then tripsinizated, and 2.5 × 10^6^ tumoroid AGS R-CDDP cells in a total volume of 0.2 ml (1:1 Matrigel^®^ Matrix, Cat#356255, Corning, USA) were injected subcutaneously into the flanks of BalbC NOD/SCID mice. Tumor growth was monitored by palpation (volume = width2 x length x π/6) as described by Lobos-González et al. [25, 26]. Mice were randomized into four different treatment groups: **Group 1** (**control**, *n* = 6) treated with NaCl 0.9% p/v; **Group 2** (**MVC**, *n* = 6) treated with MVC 10 mg/kg; **Group 3** (**CDDP**, *n* = 5) treated with CDDP 10 mg/kg; and **Group 4** (**MVC + CDDP**, *n* = 4) treated with the combination of MVC and CDDP. MVC and CDDP doses were obtained from Mencarelli et al. and Xu et al. respectively [23, 27]. The doses were administered intra-peritoneally every three days for a total of 20 days, starting three days after cell inoculation.

### Statistical analysis

All the experiments were performed in biological and technical triplicates for each condition. Data were analyzed using the GraphPad Prism 10.0.3 software (GraphPad, USA). Cell viability and cell cycle data were analyzed using a two-way ANOVA with Bonferroni post-hoc test. Cell death and RT-qPCR data were analyzed using Kruskal-Wallis with Dunn post-hoc test. Tumoroid formation was analyzed using one-way ANOVA with Tukey´s multiple comparisons post-hoc test. Mixed-effect analysis followed by Tukey’s multiple comparison test was used for tumor growth. Terminal tumor size was analyzed using one-way ANOVA with Tukey´s multiple comparisons post-hoc test. Animal survival was analyzed using Log-rank (Mantel-Cox) test.

## Results

### MVC/CDDP synergistically decreases cell viability in CDDP-resistant AGS cells

**Fig. 1:**
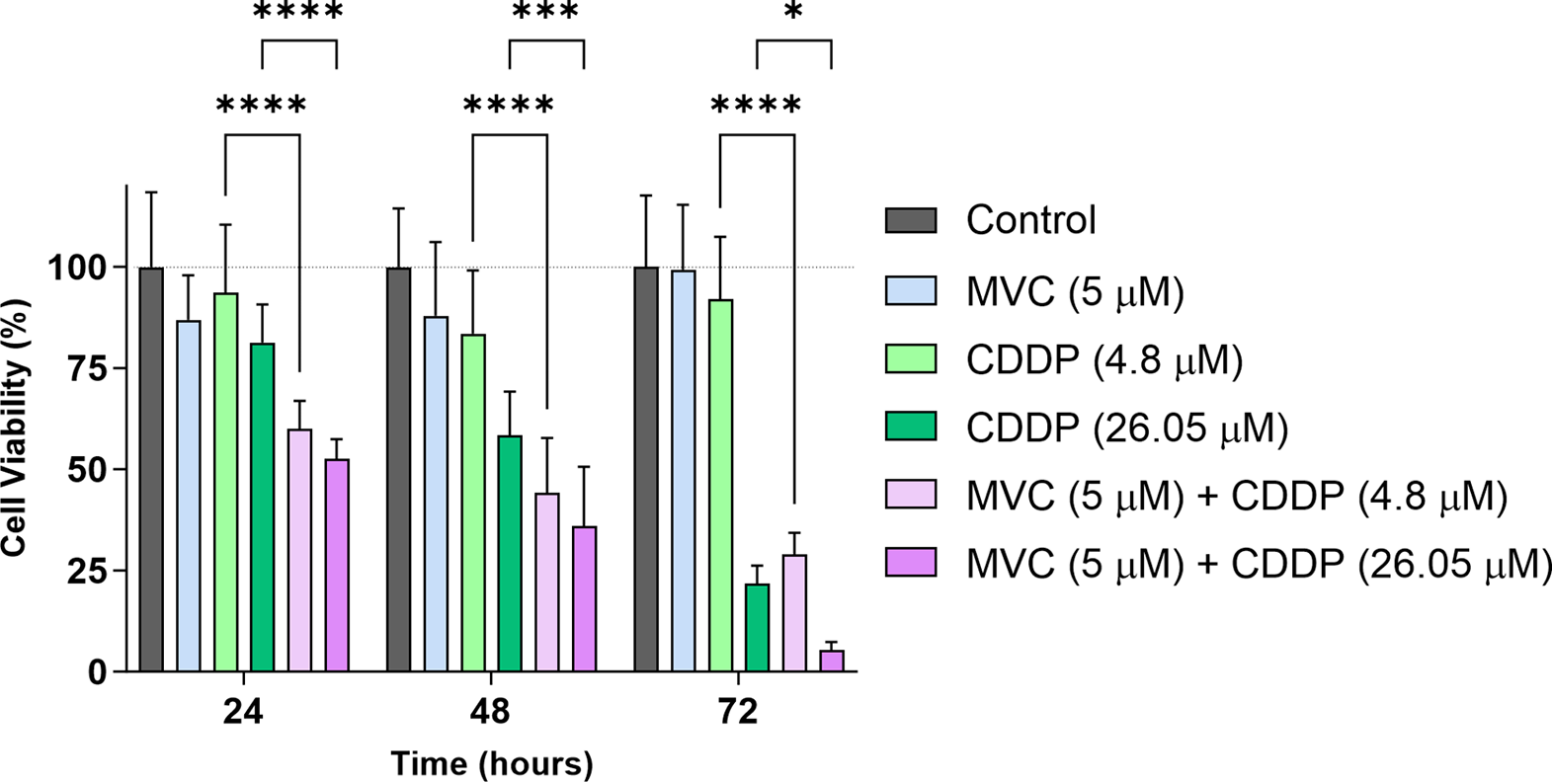
Cell Viability of AGS R-CDDP cells exposed to MVC and/or CDDP. Cells were incubated with MVC and/or CDDP for 24, 48, and 72 h, and cell viability was measured using a standard viability assay (MTT). Two-way ANOVA with Bonferroni *post-hoc* test was used to compare the groups. Values of *P* ≤ 0.05 were considered statistically significant. *P ≤ 0.05, ***P < 0.001 and ***P < 0.0001. Data were expressed as mean ± SD of three biological replicates. Maraviroc (MVC) and Cisplatin (CDDP)

MTT assays were performed in AGS R-CDDP cells exposed to MVC and/or CDDP to evaluate the effect of CCR5 inhibition on cell viability (Fig. 1). No differences in cell viability were observed between cells exposed to MVC and control cells at 24-, 48-, and 72-h post-incubation. However, a substantial decrease in cell viability was observed when MVC was combined with CDDP. MVC combined with a low concentration of CDDP (4.8 µM) triggered a reduction in cell viability at 24 (*P* < 0.0001), 48 (*P* < 0.0001), and 72 (*P* < 0.0001) h post-incubation compared to control cells. Similarly, when MVC was combined with a high concentration of CDDP (26.05 µM), cell viability was significantly reduced at all times of incubation (*P* < 0.0001) compared to control cells. As was expected, cells exposed to a high concentration of CDDP showed a viability reduction 24 (*P* < 0.01), 48 (*P* < 0.0001), and 72 h after incubation (*P* < 0.0001) compared to control cells. When MVC/CDDP combinations were compared to cells exposed to CDDP (at high or low concentrations), we observed a significant reduction of cell viability at all incubation times. The most remarkable effect in cell viability decrease was observed after 72 h of incubation with the combination of MVC and CDDP in high concentration (5.37% cell viability), which was significantly lower than the cell viability of control cells (100% cell viability) and cells exposed to a high concentration of CDDP treatment (21.74%) cell viability. A cell viability reduction was also observed among cells exposed to MVC/CDDP combinations at 72 h after incubation (*P* < 0.001).

### Dose-dependent effects of MVC/CDDP combination on cell death in CDDP-resistant AGS cells

**Fig. 2:**
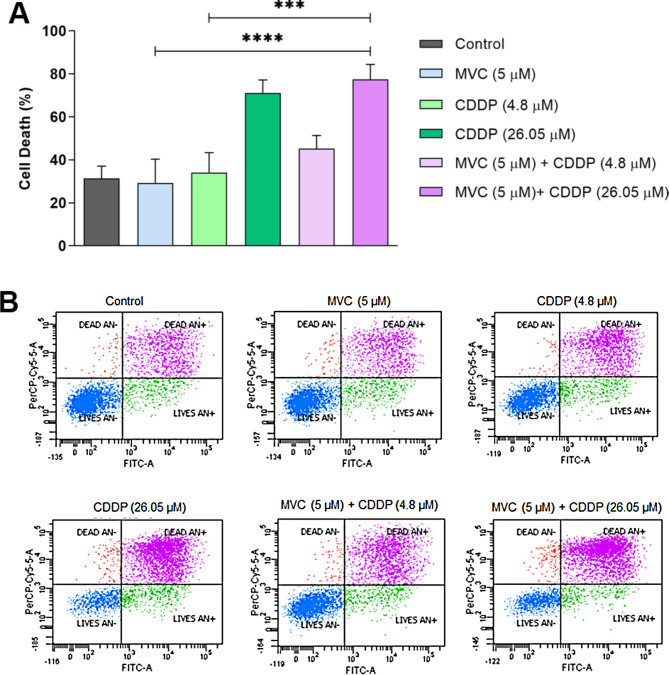
Percentage of cell death of AGS R-CDDP cells exposed to MVC and/or CDDP. The results include annexin V-positive/PI-negative cells at Q4 coordinate, annexin V/PI double-positive cells at Q2, and annexin V-negative/PI-positive cells at Q1. Cells were incubated for 72 h with MVC and/or CDDP, and the cell death was measured by flow cytometry. The fluorescence was read at maximum excitation/emission of 499⁄521 for Alexa Fluor 488 annexin V and 535⁄617 for PI. Kruskal-Wallis with Dunn *post-hoc* test was used to compare groups. Values of *P* ≤ 0.05 were considered statistically significant. ***P < 0.001 and ****P < 0.0001. Data were expressed as mean ± SD of three biological replicates. Maraviroc (MVC) and Cisplatin (CDDP)

The analysis of cell death by flow cytometry using annexin V and PI staining and representative cytometric profiles (dot plots) are shown in Fig. 2. As expected, cell death was increased in cells exposed to a high concentration of CDDP (*P* < 0.01) compared to control cells. In the same way, we observed a greater cell death in those resistant cells exposed to the combination of MVC and CDDP at high concentration in comparison with MVC (*P* < 0.0001) and CDDP low concentration (*P* < 0.001). In contrast, no differences were observed in the overall cell death percentages between cells exposed to MVC or CDDP at low concentration (4.8 µM). No increasement in cell death was observed in cells exposed to the combination of MVC and CDDP at low concentration. Therefore, the results of the MVC/CDDP combination on cell death were associated with a dose- dependent effect associated to high concentration of CDDP.

### Dose-dependent effects of MVC/CDDP combination on cell cycle progression in CDDP-resistant AGS cells

**Fig. 3:**
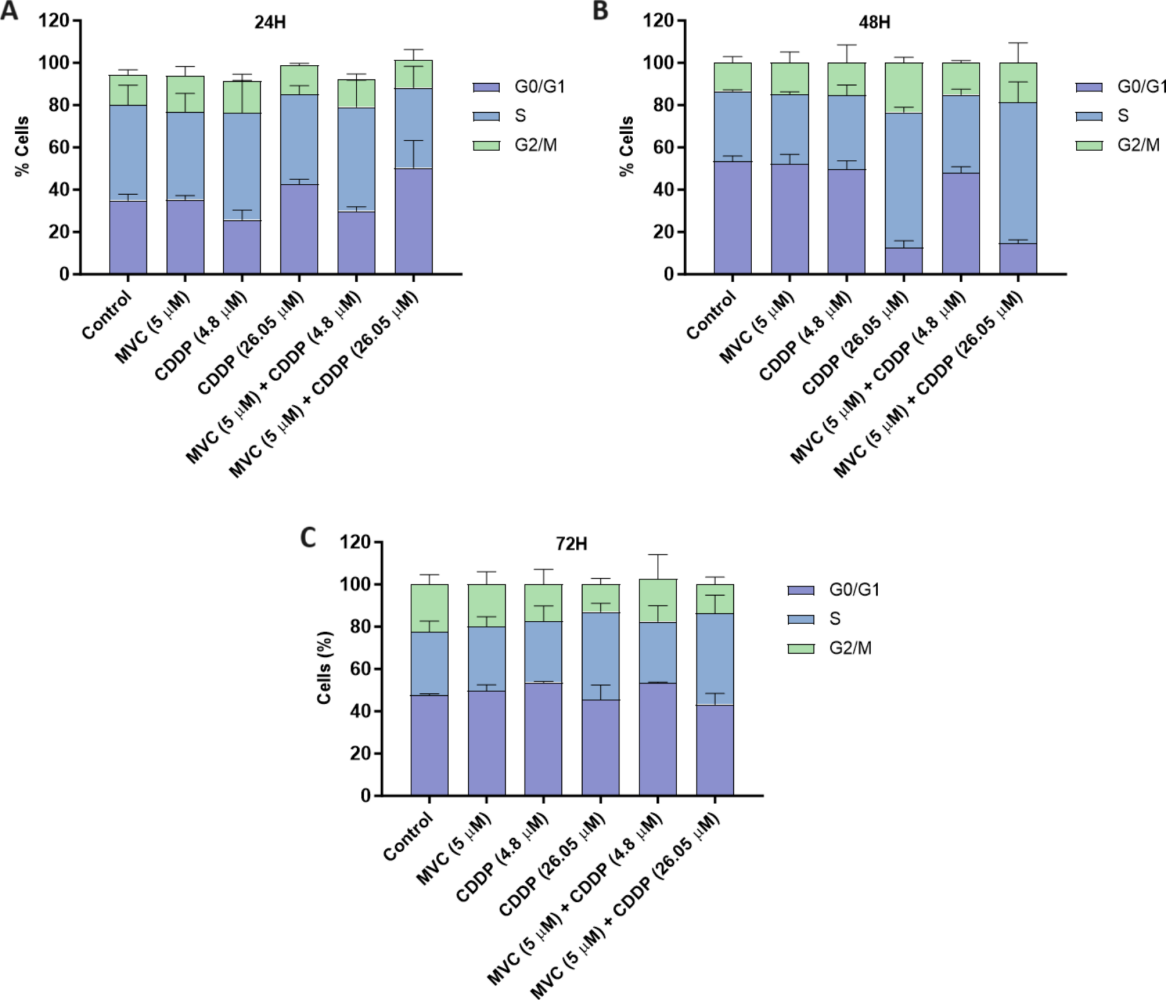
Cell cycle analysis of AGS R-CDDP cells exposed to MVC and/or CDDP. Cells were incubated with MVC and/or CDDP for **(A)** 24, **(B)** 48, and **(C)** 72 h by flow cytometry. Two-way ANOVA with Bonferroni *post-hoc* test was used to compare the groups. Data were expressed as mean ± SD of three biological replicates. Maraviroc (MVC) and Cisplatin (CDDP)

We examined the effect of CCR5 inhibition on cell cycle progression using flow cytometry analysis in AGS R-CDDP cells exposed to MVC and/or CDDP (Fig. 3). Cells exposed for 24 h to control, MVC, 4.8 µM of CDDP, and MVC/ 4.8 µM CDDP combination showed an arrest at the S phase with a concomitant decrease in the G0/G1 and G2/M phase. In contrast, cells exposed for 24 h to 26.05 µM of CDDP and MVC/26.05 µM CDDP combination showed a similar percentage of cells arrested in the G0/G1 and S phase with a decrease in the G2/M phase (Fig. 3A). When cells were exposed for 48 h to control, MVC, 4.8 µM of CDDP, and MVC/4.8 µM CDDP combination, was evident an arrest at the G0/G1 phase with a concomitant decrease in the S and G2/M phase. In contrast, cells exposed 48 h to 26.05 µM of CDDP, and MVC/26.05 µM CDDP combination, showed an arrest at the S phase with a concomitant decrease in the G0/G1 and G2/M phase (Fig. 3B). Finally, cells exposed for 72 h to control, MVC, 4.8 µM of CDDP, and MVC/4.8 µM CDDP combination showed an arrest at the G0/G1 phase with a concomitant decrease in the S and G2/M phase. In contrast, cells exposed for 72 h to 26.05 µM of CDDP, and MVC combined with 26.05 µM of CDDP showed a similar percentage of cells arrested in GO/G1 and S phase with a decrease in G2/M phase (Fig. 3C). These results indicate that MVC does not influence cell cycle progression compared to control, and the MVC/CDDP combination influences cell cycle progression on a CDDP dose-dependent manner.

### MVC/CDDP combination decreased the mRNA expression of chemokine CCL5 in CDDP-resistant AGS cells

**Fig. 4:**
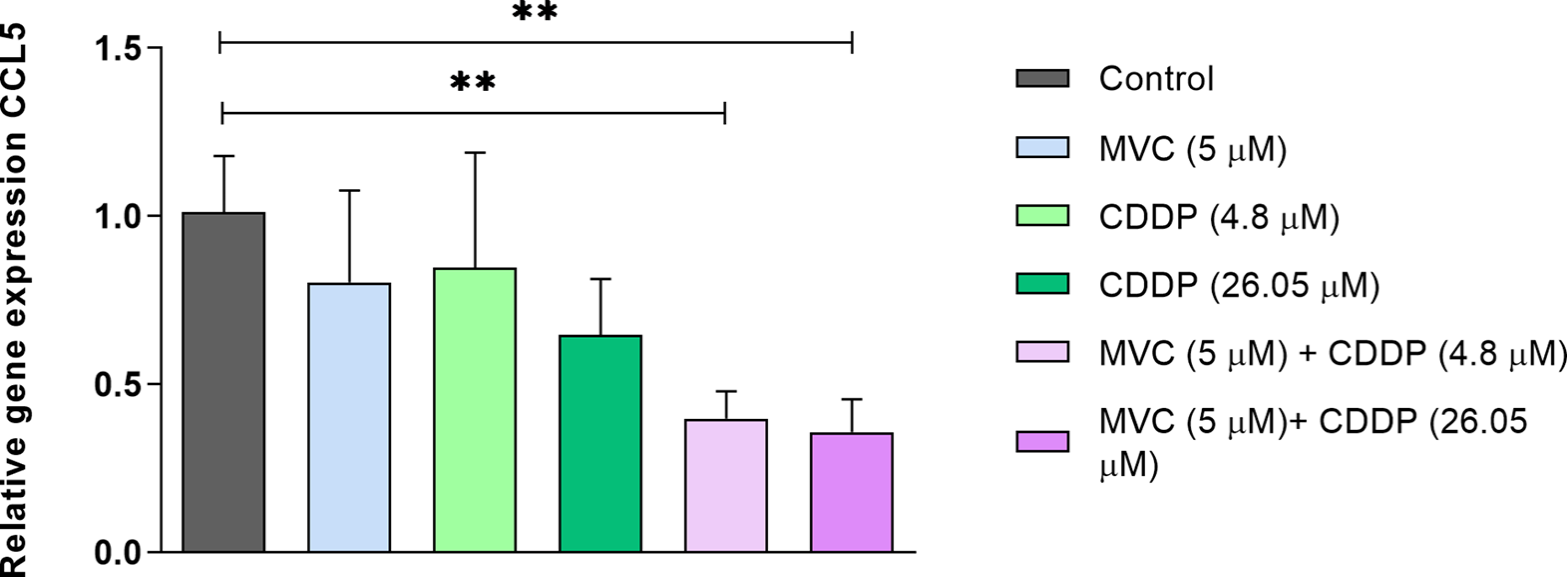
Relative expression of *CCL5* in AGS R-CDDP exposed to MVC and/or CDDP. Cells were incubated with MVC and/or CDDP for 72 h, and mRNA expression was quantified by qRT-PCR using the 2^−ΔΔCT^ method. *ACTB* was used as the reference gene. The Kruskal-Wallis test with the Dunn *post-hoc* test was used to compare groups. Values of *P* ≤ 0.05 were considered statistically significant. **P < 0.01. Data were expressed as mean ± SD of three biological replicates. Maraviroc (MVC) and Cisplatin (CDDP)

A qRT-PCR was used to evaluate the effect of CCR5 receptor inhibition on the transcriptional expression of *CCL5* (main ligand for CCR5) in AGS R-CDDP cells after 72 h of exposure to MVC and/or CDDP (Fig. 4). No differences in the *CCL5* mRNA expression were observed between cells exposed to MVC and control cells. Similarly, no differences in *CCL5* mRNA expression were observed between cells exposed to CDDP (high or low concentrations) compared to control cells. However, MVC/CDDP combinations (either low or high CDDP concentrations) evidenced a significant reduction of *CCL5* mRNA expression compared with control cells (*P* < 0.01). No differences were observed when the mRNA expression of *CCL5* was compared between cells exposed to different MVC/CDDP combinations.

### MVC/CDDP combination inhibits tumoroid formation in CDDP-resistant AGS cells

**Fig. 5:**
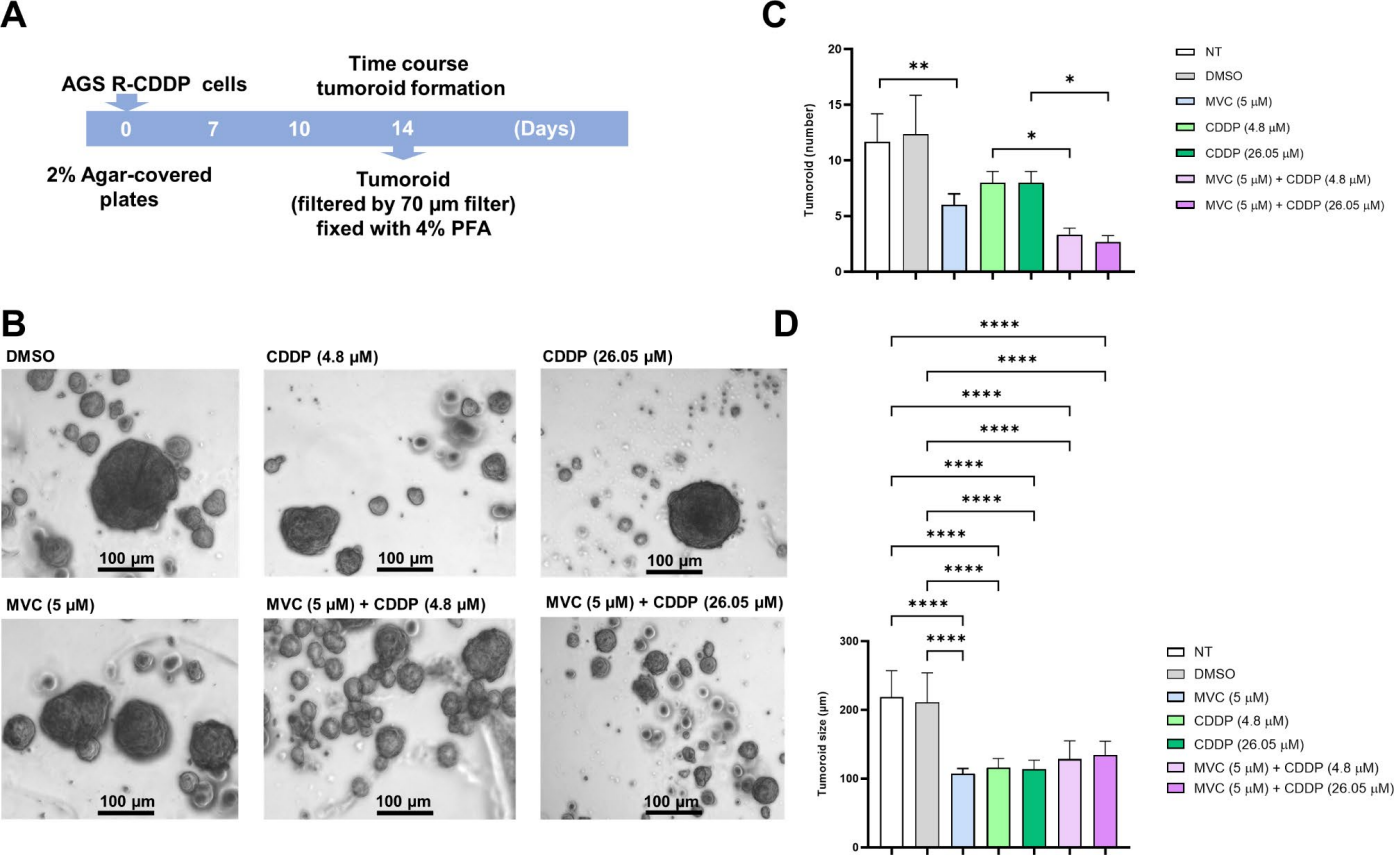
Tumoroid formation. **(A)** Scheme of development of tumoroids from AGS-RCDDP cells. **(B)** Representative images of tumoroids (*3D* tumor spheroids) on day 24. **(C)** Tumoroids number after treatments with MVC and/or CDDP. (D) Tumoroids size after treatments with MVC and/or CDDP. Tumoroids with a size > 100 μm were quantified. The size bar corresponds to 100 μm. Tumoroid formation and size were analyzed using one-way ANOVA with Tukey´s multiple comparisons post-hoc test. Values of *P* ≤ 0.05 were considered statistically significant. *P ≤ 0.05, **P < 0.01, ****P < 0.0001. Data were expressed as mean ± SD of three biological replicates. Maraviroc (MVC) and Cisplatin (CDDP)

Tumoroid formation assay using AGS R-CDDP cells exposed to MVC and/or CDDP is described in Fig. 5. A significant decrease in the number of tumoroids was observed when cells were exposed to MVC/CDDP combinations compared to cells exposed only to CDDP (*P* ≤ 0.05). AGS R-CDDP cells exposed to MVC also showed a decrease in the number of tumoroids compared to control cells. No differences were observed between controls (NT and DMSO) and CDDP. (Fig. 5C). Regarding the size of the tumoroids (Fig. 5D), the results indicate that there are differences between the controls (NT and DMSO) and the treatments with MVC and/or CDDP, with the controls being larger. However, there were no size differences among the different treatments (MVC, CDDP, and MVC/CDDP combinations).

### MVC enhances the survival rate of CDDP-treated mice

**Fig. 6:**
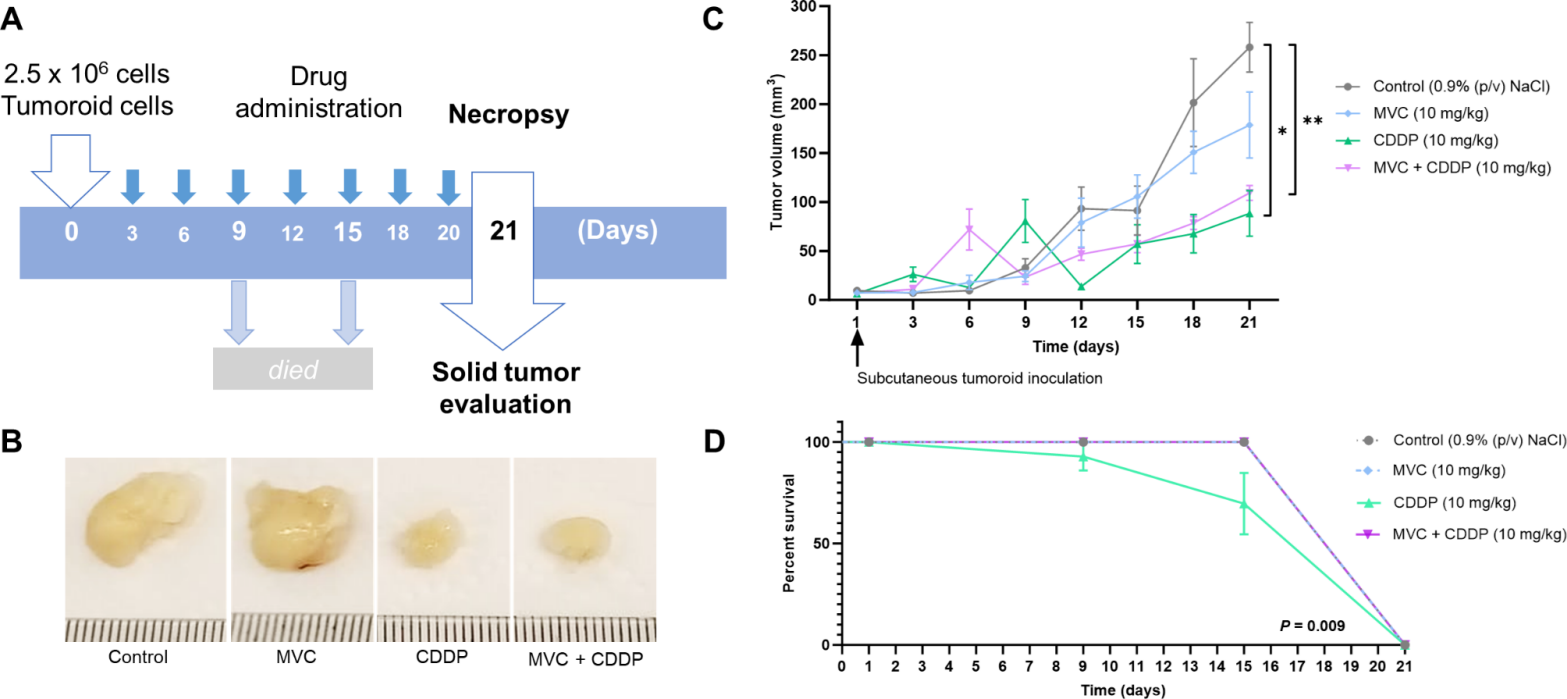
In vivo assay. **A)** Schematic subcutaneous tumor formation in BalbC NOD/SCID mice from tumoroids. **(B)** Tumor volumes of animals treated with MVC and/or CDDP were measured on day 21. **(C)** Time course of tumor formation in animals treated with MVC and/or CDDP. Mixed-effect analysis followed by Tukey’s multiple comparison test was used (*P ≤ 0.05, **P < 0.01). Data were expressed as mean ± SEM of three biological replicates) **D)** Survival chart of animals treated with MVC and/or CDDP. The Log-rank (Mantel-Cox) test was used to assess animal survival. The overlap of the survival curves for the control group, the group treated with MVC, and the group treated with the MVC/CDDP combination indicates that there were no deaths before euthanasia, in contrast to the group treated with CDDP alone. Values of *P* ≤ 0.05 were considered statistically significant. Maraviroc (MVC) and Cisplatin (CDDP)

We evaluated the progression of tumor formation following subcutaneous injections of tumoroids in mice (Fig. 6). Tumor formation was significantly reduced with CDDP treatment, both alone and in combination with MVC, compared to the control group (*P* < 0.05, *P* < 0.001, respectively). However, no differences were observed between CDDP alone and the MVC/CDDP combination in their ability to reduce subcutaneous tumor formation (Fig. 6B, C). A comparison of terminal tumor sizes between groups at day 21 is shown in Supplementary Fig. 2. More notably, the survival rate of mice treated with CDDP was markedly improved when combined with MVC (Fig. 6D). No deaths occurred in the MVC/CDDP group until euthanasia on day 21, similar to the control and MVC-only groups. In contrast, mice treated with CDDP alone had deaths on days 9 and 15, with only 50% surviving until day 21. Biochemical parameters (Supplementary Table 2) revealed increased levels of essential systemic metabolites (glucose, lactate, and creatinine) and electrolytes (sodium and chloride) in the CDDP-only group. In contrast, these parameters were reduced in the MVC/CDDP group, contributing to improved survival and quality of life in the animals.

## Discussion

Platinum chemotherapeutic agents such as CDDP are currently used to treat GC [4, 5]. Unfortunately, chemoresistance is the most important cause of treatment failure and mortality in this type of cancer [8]. The biological events that trigger or participate in resistance to therapy, laid yet unknown. In this sense, the CC chemokines subfamily has been involved in CDDP chemoresistance [11]. In fact, previously our team reported that CCL5 is up-regulated in CDDP-resistant cells, as determined by RNA-seq analysis and transcriptional validation [12]. In this regard, we continued to study CCL5/CCR5 axis in GC models of CDDP-resistance. CCR5 and its ligand CCL5 have been involved in cancer progression [13, 14]. CCR5 is a G-protein coupled transmembrane receptor that was first described as a co-receptor of HIV in immune cells [13]. Furthermore, CCR5 inhibition by Maraviroc (MVC), an allosteric and reversible inhibitor, is considered a therapeutic strategy used in clinical trials for the treatment of metastatic breast and colon cancer [13, 15]. Most studies of MVC’s antagonism over CCR5 in cancer cells have shown effects such as decreased growth, invasion, and metastasis [21, 28, 29]. However, there is scarce evidence regarding its effect on chemoresistance. We hypothesize that CCR5 inhibition by MVC might sensitize GC cells resistant to CDDP (AGS R-CDDP). Because CDDP can bind covalently to DNA forming adducts that cause DNA damage, the cell viability, cell cycle progression and cell death assays were analyzed. Furthermore, the mRNA expression of CCL5, the main CCR5 ligand, was evaluated. Finally, to evaluate the role of MVC in tumor growth and survival, *3D* tumor spheroid obtained from AGS R-CDDP cells were used to induce tumors in immune-compromised BalbC NOD/SCID mice. Our study is the first to evidence a synergistic effect of MVC and CDDP over platinum drug-resistant GC cells.

In the present study, we observed that *CCL5* mRNA expression was decreased in AGS-R cells treated with the MVC/CDDP combination. This finding suggests that *CCL5* may be regulated by an autocrine mechanism in CDDP resistance. *CCL5* expression is associated with CDDP resistance, due to the increased secretion of CC chemokines from both: autocrine manner, where the cell sends signals to itself through the secretion of ligands that bind to receptors on its own surface [30], and the tumor microenvironment (paracrine regulation) through the activation of pathways, such as Proline-rich tyrosine kinase 2 (PYK2) and Signal transducer and activator of transcription 3 (STAT3) [31, 32]. The autocrine regulation of *CCL5* can affect both the quantity available and the binding to its receptor, consequently influencing its expression. As a result, inhibiting CCR5 leads to a decrease in the expression of *CCL5* [33].

We also explored the effects of MVC and/or CDDP on cell viability, cell death and cell cycle progression. Several studies have associated MVC with decreased cell viability, proliferation, and apoptotic effects [21, 23]. Even so, in our study, MVC by itself was not capable to reduce cell viability in AGS R-CDDP cells. However, cell viability decreased when MVC was combined with CDDP (high or low concentration). Hence, the MVC/CDDP combination was significantly more effective than MVC alone. On the other hand, MVC also did not affect the cell death of AGS R-CDDP cells. We only observed an increase in cell death for cells treated with MVC/CDDP combinations, which directly correlated with the increase of CDDP concentration in the mixture. Casagrande et al.. found that MVC decreased cell viability but did not induce apoptosis-necrosis in classic Hodgkin lymphoma cells (cHL) [34]. Our results demonstrate that the synergistic effect of MVC and CDDP is unrelated to the apoptotic pathway. In addition, the combination of MVC/CDDP has been proved only in cHL, where MVC had an additive or antagonistic effect on tumor cell growth, depending on the concentrations used [34]. However, our study is the first to provide evidence of the effect of CCR5 inhibition by MVC using CDDP-resistant GC cells. We also observed a dose-dependent effect of CDDP in cell cycle progression of AGS R-CDDP cells exposed to MVC/CDDP combination, causing increased cell arrest in the GO/G1 and/or S phases, at higher concentrations of CDDP. CDDP-induced DNA damage leads to cell cycle arrest or apoptosis [35]. Previous studies, in liver and oral carcinoma cells, have shown that CDDP-induced cell cycle arrest is linked to p53 and p16 expression [36, 37]. The effect of CDDP in the S phase of chemoresistant breast cancer cells (MCF-7) revealed a decrease in apoptosis induced by the drug and an increase in the arrest of DNA synthesis [38]. In resistant cancer cells, such arrests can promote DNA repair before replication or mitosis, thereby limiting cell death by DNA-damaging agents [39] like CDDP. In this sense and considering our results demonstrate that the synergistic effect of MVC and CDDP is not related to the apoptotic pathway or to an arrest of the cell cycle in S phase, the DNA repair pathway could be associated with the re-sensitization of AGS R-CDDP cells. Jiao et al.. showed that CCR5 induces double-strand and single-strand DNA damage repair in breast cancer cells in response to DNA-damaging agents used in chemotherapy and high-dose γ-irradiation [40]. Because CCL5/CCR5 is one the central axis of activation for DNA repair from damage caused by CDDP [15] and DNA repair is one of the most important mechanisms of CDDP resistance [41–43], we hypothesized that CCR5 inhibition by MVC could enhance the action of CDDP in gastric cancer platinum-resistant cells [35].

Additionally, we used an in vitro three-dimensional (*3D*) tumor spheroid model to study the effects of MVC and CDDP on gastric cancer. This model has been developed, validated, and characterized in previous studies by our laboratory group [44–46]. The spheroid model replicates tumor-like conditions, including nutrient exposure and hypoxia [47] and overcomes *2D* culture limitations including reduced cell connections, uniform nutrient distribution, and limited differentiation [48]. Spheroids are adaptable for assessing the cytotoxic effects of treatment [49]. They enhance cell-cell and tumor microenvironment representation, which aids in tumor growth understanding by considering interactions with immune cells, stem cells, and fibroblasts [50]. Additionally, they possess cancer stem cell potential [51]. CDDP and MVC have been previously used in spheroid models [28, 34, 52–58], however, MVC/CDDP combination is novel in cancer treatment. In our study, the results of MVC/CDDP combination were superior to those with CDDP in to effectively delay the tumoroid growth. The action of MVC influenced the effect observed with the combination of tumor cells that can have chemokine receptors. These tumor cells can also secrete chemokines, which can then influence other tumor cells or components of the tumor microenvironment (TME) through paracrine signaling. Chemokines can also act on the tumor cell through autocrine signaling [11]. This type of communication has been associated with both tumor growth and development of chemoresistance [59]. MVC would be inhibiting the interaction between CCL5 and its receptor. Therefore, the activation of intracellular signaling cascades such as Phosphoinositide 3-kinase (PI3K), Mitogen-activated protein kinase (MAPK), and the Janus kinase signal transducer and activator of transcription (JAK-STAT) pathway, which are involved in carcinogenesis, tumor growth, and regulation of other cytokines, is inhibited. This would lead to a decrease in the development of spheroids [13].

Furthermore, in our study, we used BalbC NOD/SCID mice to study the effects of MVC and CDDP in a more complex model. Considering that for the animal model the tumoroids were trypsinized, and the cells constituting them were injected into the animals, the results should be interpreted as the survival of the tumoroid-derived cells, which possess cancer stem cell potential [51] and are exposed to the different treatments with MVC and/or CDDP. We found no significant difference in tumor growth in animals treated with CDDP or in combination with MVC. However, the survival rate and biochemical parameters were significantly improved in animals treated with CDDP/MVC combination; glucose, lactate, creatinine, sodium, and chloride levels were decreased compared to animals only treated with CDDP, and hematocrit levels were found between the normal range, in comparison with animals exposed to only CDDP treatment. Therefore, MVC/CDDP combination, reduced the side effects of CDDP. In contrast, animals treated with CDDP showing decreased survival rate and abnormal biochemical parameters, both associated with CDDP side effects. In this group, we observed elevated creatinine levels and electrolytes related to CDDP nephrotoxicity. These findings are consistent with the fact that one-third of patients treated with CDDP experience nephrotoxicity levels, manifesting decreased glomerular filtration rates, elevated serum creatinine, and electrolyte imbalance [60]. We also observed a decrease in hematocrits (Hct) and an increase in lactate, associated with anemia caused by CDDP therapy [61]. Other changes were also observed in presence of CDDP, such as an increase in glucose and chloride levels. Yadav et al.. demonstrated that CDDP increased glucose and glycated hemoglobin (Gly Hb) levels in Wistar rats after a single dose of 10 mg/kg [62]. They showed that platinum drugs can cause hyperglycemia in animal models. Similarly, Guo et al. demonstrated that carboplatin administration could cause transient hyperglycemia in rats, which might occur by the combination of glucagon accumulation caused by the decrease in islet cell secretion [63]. The increased chloride levels may be due to the entry of CDDP into the cell, where the chloride ligands are replaced by water molecules, which could explain the chloride accumulation [64].

The mechanisms by which MVC improves survival rate and decreases CDDP side effects in GC animal models have not yet been studied. However, the anticancer potential of MVC has been demonstrated in NOD/SCID mice with GC cells (MKN-45) injected into the peritoneal cavity [23]. Mencarelli et al. showed that MVC reduced the number and total volume of peritoneal and mesenteric nodules and increased median survival time. Furthermore, administration of MVC reduced tumor burden and caused extensive intratumoral necrosis in mice implanted with MKN45 and MKN74 cells. The authors associated these results with regulating the expression of several genes, including interleukin 10 receptor subunit beta (IL-10RB), Mesenchymal-epithelial transition factor (MET), FAT atypical cadherin 1 (FAT1), Nucleoside Diphosphate Kinase A (NME1), and Lymphotoxin beta (LTB) [23]. A phase I trial named MARACON (NCT01736813) showed that inhibition of CCR5 leads to an antitumoral activation of macrophages, affecting the composition of immune cell infiltrates. The trial demonstrated that this inhibition leads to the repolarization of tumor-associated macrophages toward an M1-like phenotype, promoting a tumor-inhibiting immune milieu in patients with liver metastases from advanced refractory colorectal cancer [29]. Clinical reports about MVC use have shown that the drug is well tolerated and does not compromise immune responses [65].

To date, there is no precedent regarding the mechanism by which MVC and CDDP exert their effects on drug chemoresistance. However, drawing from the findings of Tupova et al. who have demonstrated interactions between MVC and ATP-binding cassette sub-family B member 1 (ABCB1), ATP-binding cassette super-family G member 2 (ABCG2), and ATP-Binding Cassette Sub-Family C Member 2 (ABCC2) transporters, we presume that MVC’s mode of action may extend beyond the exclusive inhibition of CCR5. This raises the intriguing possibility that MVC could also bind to these transporter proteins. From this perspective, it is speculated that if MVC were able to bind to these efflux transporters, it could potentially lead to a prolonged intracellular retention of CDDP [66].

## Conclusions

In in vitro studies, the combination of MVC/CDDP decreases cell viability, increasing sensitivity to this platinum drug. In the *3D* spheroid model, the combination inhibits growth. In the murine model, the MVC/CDDP combination results in increased survival and reduces the adverse effects associated with CDDP monotherapy. The MVC/CDDP combination shows promise as an adjunctive therapy for gastric cancer. Future studies focused on tumor invasion, metastasis, and clinical trials should be conducted to confirm the synergistic effect of the combination of MVC and CDDP.

## Electronic supplementary material

Below is the link to the electronic supplementary material.

Supplementary Material 1

## Data Availability

The data generated in the present study may be requested from the corresponding author.

## Declarations

### Patents

These results of this study are protected by a patent application filed with the Instituto Nacional de Propiedad Industrial (INAPI) under Application number PCT/CL2024/050006.

## Abbreviations

ABCB1: ATP-binding Cassette Sub-Family B member 1
ABCC2: ATP-Binding Cassette Sub-Family C Member 2
ABCG2: ATP-binding Cassette Sub-Family G member 2
AGS R-CDDP: CDDP-resistant AGS cells
CDDP: Cisplatin
cHL: Hodgkin lymphoma cells
CCL5: C-C Motif Chemokine Ligand 5
CCR5: C-C chemokine receptor type 5
FAT1: Fat atypical cadherin 1
GC: Gastric cancer
Gly Hb: Glycated hemoglobin
HIV-1: Human immunodeficiency virus type 1
IL-10RB: Interleukin 10 receptor subunit beta
JAK-STAT: Janus kinase signal transducer and activator of transcription pathway
LTB: Lymphotoxin beta
MAPK: Mitogen-activated protein kinase
MEGM: Mammary Epithelial Cell Growth culture medium
MET: Mesenchymal-epithelial transition factor
MVC: Maraviroc
NME1: Nucleoside Diphosphate Kinase A
PI: Propidium iodide
PI3K: Phosphoinositide 3-kinase
Pyk2: Proline-rich tyrosine kinase 2
RANTES: Regulated upon Activation, Normal T-cell Expressed and Secreted
STAT3: Signal transducer and activator of transcription 3
3D: Three-dimensional

## References

[1] Sung H, Ferlay J, Siegel RL, Laversanne M, Soerjomataram I, Jemal A, et al. Global Cancer statistics 2020: GLOBOCAN estimates of incidence and Mortality Worldwide for 36 cancers in 185 countries. CA Cancer J Clin. 2021;71(3):209–49. 33538338 10.3322/caac.21660

[2] Marin JJG, Al-Abdulla R, Lozano E, Briz O, Bujanda L, Banales M. Mechanisms of resistance to Chemotherapy in Gastric Cancer. Anticancer Agents Med Chem. 2016;16(3):318–34. 26234359 10.2174/1871520615666150803125121

[3] Zhang D, Fan D. New insights into the mechanisms of gastric cancer multidrug resistance and future perspectives. Future Oncol. 2010;6:527–37. 20373867 10.2217/fon.10.21

[4] DIgklia A, Wagner AD. Advanced gastric cancer: current treatment landscape and future perspectives. World J Gastroenterol. 2016. 10.3748/wjg.v22.i8.2403 PMC4768187 26937129

[5] Keehn RJ, Higgins GA. Chemotherapy for gastric cancer. Lancet. 1981;1(8215):323. 6109953 10.1016/s0140-6736(81)91928-0

[6] Wang D, Lippard SJ. Cellular processing of platinum anticancer drugs. Nat Rev Drug Discov. 2005;4(4):307–20. 15789122 10.1038/nrd1691

[7] Shi WJ, Gao JB. Molecular mechanisms of chemoresistance in gastric cancer. World J Gastrointest Oncol. 2016;8(9):673. 27672425 10.4251/wjgo.v8.i9.673 PMC5027022

[8] Huang H, Han Y, Zhang C, Wu J, Feng J, Qu L, et al. HNRNPC as a candidate biomarker for chemoresistance in gastric cancer. Tumour Biol. 2016;37(3):3527–34. 26453116 10.1007/s13277-015-4144-1

[9] Chen SH, Chang JY. New insights into mechanisms of cisplatin resistance: from tumor cell to microenvironment. Int J Mol Sci. 2019;20(17). 10.3390/ijms20174136 PMC6747329 31450627

[10] Mora Y, Reyes ME, Zanella L, Mora B, Buchegger K, Ili C, et al. Resistance to platinum-based cancer drugs: a special focus on epigenetic mechanisms. Pharmacogenomics. 2021;22(12):777–90. 34281355 10.2217/pgs-2021-0020

[11] Reyes ME, de La Fuente M, Hermoso M, Ili CG, Brebi P. Role of CC chemokines Subfamily in the platinum drugs Resistance Promotion in Cancer. Front Immunol. 2020. 10.3389/fimmu.2020.00901 PMC7243460 32499779

[12] Mora-Lagos B, Cartas-Espinel I, Riquelme I, Parker AC, Piccolo SR, Viscarra T et al. Functional and transcriptomic characterization of cisplatin-resistant AGS and MKN-28 gastric cancer cell lines. PLoS ONE. 2020. 10.1371/journal.pone.0228331 PMC6986722 31990955

[13] Aldinucci D, Borghese C, Casagrande N. The CCL5/CCR5 Axis in Cancer Progression. Cancers (Basel). 2020;12(7). 10.3390/cancers12071765 PMC7407580 32630699

[14] Ryu H, Baek S, Moon J, Jo I, Kim N, Lee H. CC motif chemokine receptors in gastric cancer (review). Mol Clin Oncol. 2017. 10.3892/mco.2017.1470 PMC5738695 29285394

[15] Jiao X, Nawab O, Patel T, Kossenkov AV, Halama N, Jaeger D et al. Recent advances targeting CCR5 for cancer and its role in immuno-oncology. Cancer Res. 2019. 10.1158/0008-5472.CAN-19-1167 PMC6810651 31292161

[16] Aldinucci D, Casagrande N. Inhibition of the CCL5/CCR5 Axis against the Progression of Gastric Cancer. Int J Mol Sci [Internet]. 2018;19(5):1477. https://pubmed.ncbi.nlm.nih.gov/29772686 10.3390/ijms19051477 PMC5983686 29772686

[17] Mukaida N, Tanabe Y, Baba T. Chemokines as a conductor of bone marrow microenvironment in chronic myeloid leukemia. Int J Mol Sci. 2017;18(8):1824. 28829353 10.3390/ijms18081824 PMC5578209

[18] Velasco-velazquez M, Jiao X, Fuente MD, La, Pestell TG. CCR5 antagonist blocks metastasis of basal breast Cancer cells CCR5 antagonist blocks metastasis of basal breast Cancer cells. 2012;(December 2015). 10.1158/0008-5472.CAN-11-3917 22637726

[19] Cambien B, Richard-Fiardo P, Karimdjee BF, Martini V, Ferrua B, Pitard B, et al. CCL5 neutralization restricts Cancer Growth and Potentiates the Targeting of PDGFRβ in Colorectal Carcinoma. PLoS ONE. 2011;6(12):e28842. 22205974 10.1371/journal.pone.0028842 PMC3243667

[20] Yang T, Chen M, Yang X, Zhang X, Zhang Z, Sun Y, et al. Down-regulation of KLF5 in cancer-associated fibroblasts inhibit gastric cancer cells progression by CCL5/CCR5 axis. Cancer Biol Ther. 2017;18(10):806–15. 28934010 10.1080/15384047.2017.1373219 PMC5678703

[21] Pervaiz A, Zepp M, Mahmood S, Ali DM, Berger MR, Adwan H. CCR5 blockage by maraviroc: a potential therapeutic option for metastatic breast cancer. Cell Oncol. 2019;42(1):93–106. 10.1007/s13402-018-0415-3 PMC12994360 30456574

[22] Ding H, Zhao L, Dai S, Li L, Wang F, Shan B. CCL5 secreted by tumor associated macrophages may be a new target in treatment of gastric cancer. Biomed Pharmacotherapy. 2016. 10.1016/j.biopha.2015.12.004 26796278

[23] Mencarelli A, Graziosi L, Renga B, Cipriani S, D’Amore C, Francisci D et al. CCR5 antagonism by maraviroc reduces the potential for gastric cancer cell dissemination. Transl Oncol. 2013. 10.1593/tlo.13499 PMC3890714 24466382

[24] Durán-Jara E, del Campo M, Gutiérrez V, Wichmann I, Trigo C, Ezquer M, et al. Lactadherin immunoblockade in small extracellular vesicles inhibits sEV-mediated increase of pro-metastatic capacities. Biol Res. 2024;57(1):1. 38173019 10.1186/s40659-023-00477-8 PMC10763369

[25] Lobos-González L, Aguilar L, Diaz J, Diaz N, Urra H, Torres VA et al. E-cadherin determines Caveolin-1 tumor suppression or metastasis enhancing function in melanoma cells. Pigment Cell Melanoma Res [Internet]. 2013;26(4):555–70. https://onlinelibrary.wiley.com/doi/abs/10.1111/pcmr.12085 10.1111/pcmr.12085 PMC3695072 23470013

[26] Lobos-Gonzalez L, Aguilar-Guzmán L, Fernandez JG, Muñoz N, Hossain M, Bieneck S et al. Caveolin-1 is a risk factor for postsurgery metastasis in preclinical melanoma models. Melanoma Res [Internet]. 2014;24(2). https://journals.lww.com/melanomaresearch/fulltext/2014/04000/caveolin_1_is_a_risk_factor_for_postsurgery.3.aspx 10.1097/CMR.0000000000000046 PMC3999137 24500501

[27] Xu H, Bin, Shen FM, Lv QZ. Celecoxib enhanced the cytotoxic effect of cisplatin in chemo-resistant gastric cancer xenograft mouse models through a cyclooxygenase-2-dependent manner. Eur J Pharmacol. 2016. 10.1016/j.ejphar.2016.02.035 26879869

[28] Singh SK, Mishra MK, Eltoum IEA, Bae S, Lillard JW, Singh R. CCR5/CCL5 axis interaction promotes migratory and invasiveness of pancreatic cancer cells. Sci Rep. 2018;8(1):1323. 29358632 10.1038/s41598-018-19643-0 PMC5778036

[29] Halama N, Zoernig I, Berthel A, Grabe N, Falk CS, Jaeger D, et al. Tumoral Immune Cell Exploitation in Colorectal Cancer metastases can be targeted effectively by Anti-CCR5 therapy in Cancer patients Article Tumoral Immune Cell Exploitation in Colorectal Cancer metastases can be targeted effectively by Anti-CCR5 therapy. Cancer Cell. 2016;29(4):587–601. 27070705 10.1016/j.ccell.2016.03.005

[30] Jones VS, Huang RY, Chen LP, Chen ZS, Fu L, Huang RP. Cytokines in cancer drug resistance: cues to new therapeutic strategies. Biochim Biophys Acta Rev Cancer. 2016;1865(2):255–65. 10.1016/j.bbcan.2016.03.005 26993403

[31] Pasquier J, Gosset M, Geyl C, Hoarau-Véchot J, Chevrot A, Pocard M et al. CCL2/CCL5 secreted by the stroma induce IL-6/PYK2 dependent chemoresistance in ovarian cancer. Mol Cancer. 2018. 10.1186/s12943-018-0787-z PMC5817856 29455640

[32] Zhou B, Sun C, Li N, Shan W, Lu H, Guo L, et al. Cisplatin-induced CCL5 secretion from CAFs promotes cisplatin-resistance in ovarian cancer via regulation of the STAT3 and PI3K/Akt signaling pathways. Int J Oncol. 2016;48(5):2087–97. 26983899 10.3892/ijo.2016.3442

[33] Jones VS, Huang RY, Chen LP, Chen ZS, Fu L, Huang RP. Cytokines in cancer drug resistance: cues to new therapeutic strategies. Biochimica et Biophysica Acta - Reviews on Cancer; 2016. 10.1016/j.bbcan.2016.03.005 26993403

[34] Casagrande N, Borghese C, Visser L, Mongiat M, Colombatti A, Aldinucci D. CCR5 antagonism by maraviroc inhibits hodgkin lymphoma microenvironment interactions and xenograft growth. Haematologica. 2019. 10.3324/haematol.2018.196725 PMC6395337 30309853

[35] Rocha CRR, Silva MM, Quinet A, Cabral-neto JB, Menck FMC. DNA repair pathways and cisplatin resistance: an intimate relationship. Clinics. 2018. 10.6061/clinics/2018/e478s PMC6113849 30208165

[36] Wang P, Cui J, Wen J, Guo Y, Zhang L, Chen X. Cisplatin induces HepG2 cell cycle arrest through targeting specific long noncoding RNAs and the p53 signaling pathway. Oncol Lett. 2016;12(6):4605–12. 28105167 10.3892/ol.2016.5288 PMC5228559

[37] Yip HT, Chopra R, Chakrabarti R, Veena MS, Ramamurthy B, Srivatsan ES, et al. Cisplatin-Induced growth arrest of Head and Neck Cancer cells correlates with increased expression of p16 and p53. Arch Otolaryngol Head Neck Surg. 2006;132(3):317. 16549753 10.1001/archotol.132.3.317

[38] Xu ZY, Loignon M, Han FY, Panasci L, Aloyz R. Xrcc3 induces cisplatin resistance by stimulation of Rad51-related recombinational repair, S-phase checkpoint activation, and reduced apoptosis. J Pharmacol Exp Ther. 2005;314(2):495–505. 15843498 10.1124/jpet.105.084053

[39] Li Lya, Guan Y di, Chen X, sha, Yang J ming, Cheng Y. DNA Repair Pathways in Cancer Therapy and Resistance. Front Pharmacol. 2021;11. 10.3389/fphar.2020.629266 PMC7898236 33628188

[40] Jiao X, Velasco-Velazquez MA, Wang M, Li Z, Rui H, Peck AR et al. CCR5 governs DNA damage repair and breast cancer stem cell expansion. Cancer Res. 2018. 10.1158/0008-5472.CAN-17-0915 PMC6331183 29358169

[41] Holohan C, Van Schaeybroeck S, Longley DB, Johnston PG. Cancer drug resistance: an evolving paradigm. Nat Rev Cancer. 2013;13(10):714–26. 24060863 10.1038/nrc3599

[42] Mansoori B, Mohammadi A, Davudian S, Shirjang S, Baradaran B. The different mechanisms of Cancer Drug Resistance: a brief review. Adv Pharm Bull. 2017;7(3):339–48. 29071215 10.15171/apb.2017.041 PMC5651054

[43] Housman G, Byler S, Heerboth S, Lapinska K, Longacre M, Snyder N, et al. Drug resistance in cancer: an overview. Cancers. 2014;6:1769–92. 25198391 10.3390/cancers6031769 PMC4190567

[44] Sandoval-Bórquez A, Polakovicova I, Carrasco-Véliz N, Lobos-González L, Riquelme I, Carrasco-Avino G et al. MicroRNA-335-5p is a potential suppressor of metastasis and invasion in gastric cancer. Clin Epigenetics [Internet]. 2017;9(1):114. 10.1186/s13148-017-0413-8 10.1186/s13148-017-0413-8 PMC5645854 29075357

[45] Borgna V, Lobos-González L, Guevara F, Landerer E, Bendek M, Ávila R, et al. Targeting antisense mitochondrial noncoding RNAs induces bladder cancer cell death and inhibition of tumor growth through reduction of survival and invasion factors. J Cancer. 2020;11(7):1780–91. 32194789 10.7150/jca.38880 PMC7052861

[46] Ortega L, Lobos-González L, Reyna-Jeldes M, Cerda D, De la Fuente-Ortega E, Castro P, et al. The Ω-3 fatty acid docosahexaenoic acid selectively induces apoptosis in tumor-derived cells and suppress tumor growth in gastric cancer. Eur J Pharmacol. 2021;896:173910. 33508285 10.1016/j.ejphar.2021.173910

[47] Nunes AS, Barros AS, Costa EC, Moreira AF, Correia IJ. 3D tumor spheroids as in vitro models to mimic in vivo human solid tumors resistance to therapeutic drugs. Biotechnol Bioeng. 2019;116(1):206–26. 30367820 10.1002/bit.26845

[48] Pinto B, Henriques AC, Silva PMA, Bousbaa H. Three-Dimensional spheroids as in Vitro Preclinical models for Cancer Research. Pharmaceutics. 2020;12(12):1186. 33291351 10.3390/pharmaceutics12121186 PMC7762220

[49] Zanoni M, Piccinini F, Arienti C, Zamagni A, Santi S, Polico R, et al. 3D tumor spheroid models for in vitro therapeutic screening: a systematic approach to enhance the biological relevance of data obtained. Sci Rep. 2016;6(1):19103. 26752500 10.1038/srep19103 PMC4707510

[50] Zhu Y, Kang E, Wilson M, Basso T, Chen E, Yu Y, et al. 3D Tumor Spheroid and Organoid to Model Tumor Microenvironment for Cancer Immunotherapy. Organoids. 2022;1(2):149–67.

[51] Abbasi-Malati Z, Khanicheragh P, Narmi MT, Mardi N, Khosrowshahi ND, Hiradfar A, et al. Tumoroids, a valid preclinical screening platform for monitoring cancer angiogenesis. Stem Cell Res Ther. 2024;15(1):267. 39183337 10.1186/s13287-024-03880-4 PMC11346257

[52] Singh SK, Mishra MK, Rivers BM, Gordetsky JB, Bae S, Singh R. Biological and clinical significance of the CCR5/CCL5 Axis in Hepatocellular Carcinoma. Cancers (Basel). 2020;12(4):883. 32260550 10.3390/cancers12040883 PMC7226629

[53] DiNatale A, Kaur R, Qian C, Zhang J, Marchioli M, Ipe D, et al. Subsets of cancer cells expressing CX3CR1 are endowed with metastasis-initiating properties and resistance to chemotherapy. Oncogene. 2022;41(9):1337–51. 34999735 10.1038/s41388-021-02174-w PMC8941631

[54] Novak M, Koprivnikar Krajnc M, Hrastar B, Breznik B, Majc B, Mlinar M, et al. CCR5-Mediated signaling is involved in Invasion of Glioblastoma Cells in its Microenvironment. Int J Mol Sci. 2020;21(12):4199. 32545571 10.3390/ijms21124199 PMC7352708

[55] Azharuddin M, Roberg K, Dhara AK, Jain MV, Darcy P, Hinkula J, et al. Dissecting multi drug resistance in head and neck cancer cells using multicellular tumor spheroids. Sci Rep. 2019;9(1):20066. 31882620 10.1038/s41598-019-56273-6 PMC6934860

[56] Panneerselvam J, Mohandoss P, Patel R, Gillan H, Li M, Kumar K, et al. DCLK1 regulates Tumor Stemness and Cisplatin Resistance in Non-small Cell Lung Cancer via ABCD-Member-4. Mol Ther Oncolytics. 2020;18:24–36. 32637578 10.1016/j.omto.2020.05.012 PMC7321820

[57] Raghavan S, Mehta P, Horst EN, Ward MR, Rowley KR, Mehta G. Comparative analysis of tumor spheroid generation techniques for differential *in vitro* drug toxicity. Oncotarget. 2016;7(13):16948–61. 26918944 10.18632/oncotarget.7659 PMC4941362

[58] Li C, Qiu J, Xue Y. Low-dose Diosbulbin-B (DB) activates tumor-intrinsic PD-L1/NLRP3 signaling pathway mediated pyroptotic cell death to increase cisplatin-sensitivity in gastric cancer (GC). Cell Biosci. 2021;11(1):38. 33579380 10.1186/s13578-021-00548-x PMC7881658

[59] Greten FR, Grivennikov SI. Inflammation and Cancer: triggers, mechanisms, and consequences. Vol. 51, immunity. Cell; 2019. pp. 27–41. 10.1016/j.immuni.2019.06.025 PMC6831096 31315034

[60] Pabla N, Dong Z. Cisplatin nephrotoxicity: Mechanisms and renoprotective strategies. Kidney Int [Internet]. 2008;73(9):994–1007. https://www.sciencedirect.com/science/article/pii/S0085253815531244 10.1038/sj.ki.5002786 18272962

[61] Hrushesky PAWANDWJ. Cisplatin-associated anemia: an erythropoietin deficiency syndrome. J Clin Invest [Internet]. 1995;95(4):1650–9. 10.1172/JCI117840 10.1172/JCI117840 PMC295669 7706473

[62] Yadav YC. Effect of cisplatin on pancreas and testes in Wistar rats: biochemical parameters and histology. Heliyon. 2019;5(8):e02247. 31453403 10.1016/j.heliyon.2019.e02247 PMC6700420

[63] Guo Y, Zeng H, Chang X, Wang C, Cui H. Additional dexamethasone in chemotherapies with carboplatin and paclitaxel could reduce the impaired glycometabolism in rat models. BMC Cancer. 2018;18(1):81. 29338697 10.1186/s12885-017-3917-x PMC5769515

[64] Trimmer E, Essigmann JE. Cisplatin. In: Ballou DP, editor. Essays in Biochemistry Metalloproteins. Princeton University Press; 1999.

[65] Jiao X, Velasco-Velazquez MA, Wang M, Li Z, Rui H, Peck AR, et al. CCR5 governs DNA damage repair and breast cancer stem cell expansion. Cancer Res. 2018;78(7):1657–71. 29358169 10.1158/0008-5472.CAN-17-0915 PMC6331183

[66] Tupova L, Ceckova M, Ambrus C, Sorf A, Ptackova Z, Gaborik Z. Interactions between Maraviroc and the ABCB1, ABCG2, and ABCC2 transporters : an important role in Transplacental Pharmacokinetics. 2019;1(September):954–60. 10.1124/dmd.119.087684 31266750

