# Supplementary material for: Maraviroc/cisplatin combination inhibits gastric cancer tumoroid growth and improves mice survival
Source: Biol Res. 2025 Jan 18;58:4. doi: 10.1186/s40659-024-00581-3 (PMC11748569; doi:10.1186/s40659-024-00581-3)
Supplement: Supplementary file 1 — Supplementary Material 1 [file 40659_2024_581_MOESM1_ESM.docx]

Supplementary Material

**MARAVIROC/CISPLATIN COMBINATION INHIBITS GASTRIC CANCER TUMOROID GROWTH AND IMPROVES MICE SURVIVAL**

Bárbara Mora-Lagos^†^, María Elena Reyes^†^, Lorena Lobos-Gonzalez, Matías del Campo, Kurt Buchegger, Louise Zanella, Ismael Riquelme, Carmen Gloria Ili*, Priscilla Brebi*.

| **Gene** | **Primer sequence 5'-3'** | **Amplicon (bp)** |
| --- | --- | --- |
| *ACTB* | Fw ATCATTGCTCCTCCTGAGC | 107 |
|  | Rv ACTCCTGCTTGCTGATCCAC |  |
| *CCL5* | Fw TGGGTTCGGGAGTACATCAA | 162 |
|  | Rv GTAGAATCTGGGCCCTTCAA |  |

# Supplementary Table 1. Sequences of oligonucleotides used in this study.

# Supplementary Figure 1. Tumoroid standardization. Tumorigenic assay was developed to compare the tumoroid formation rate between AGS R-CDDP and AGS WT cells exposed to Maraviroc (MVC) and/or Cisplatin (CDDP). AGS R-CDDP showed a significantly higher tumoroid formation rate than AGS WT. Tumoroid formation was analyzed using Mann-Whitney test. Values of P≤0.05 were considered statistically significant. *P≤0.05, **P<0.01.

**Supplementary Figure 2. Terminal tumor size (day 21)**. Terminal tumor size comparison between Control, Maraviroc (MVC), Cisplatin (CDDP) and MVC/CDDP groups. Tumor was decreased with the use of CDDP alone (P<0.001) and in combination with MVC (P<0.001) compared to the control group. Terminal tumor size was analyzed using one-way ANOVA with Tukey´s multiple comparisons post-hoc test. Values of P≤0.05 were considered statistically significant. **P<0.01.

| **Samples** | **Glucose mg/dL**  **120-250** | **Lactate**  **0,5 – 2,2 mmol/L** | **Creatinine mg/dL**  **0,4 a 1,5 mg/dL** | **Na**  **140-156 mmol/L** | **K**  **4,3-5,2**  **mmol/L** | **Ca**  **0,2-0,4**  **mmol/L** | **Cl**  **100-106 mmol/L** | **cTCO2**  **mmol/L** | **Hct**  **10-75 %** | **cHgb**  **3-25**  **g/dL** | **BE(b)**  **mmol/L** |
| --- | --- | --- | --- | --- | --- | --- | --- | --- | --- | --- | --- |
| Control R1 | 190 | 2.18 | 1.3 | 152 | 6,2 | ≤0,25 | 134 | 13,0 | 15 | 5,1 | -16.8 |
| Control R2 | 172 | 1.23 | 0.9 | 148 | 2.9 | 0.35 | 101 | 12.1 | 18 | 5 | -18 |
| Control R3 | 145 | 1.1 | 1.2 | 141 | 2.8 | 0.33 | 100 | 12.2 | 13 | 9.6 | -12 |
| Control R4 | 198 | 1.68 | 0.8 | 156 | 3,8 | ≤0,25 | 102 | 12 | 17 | Cnc | -17.8 |
| Control R5 | 165 | 0.64 | 0.75 | 140 | 3,1 | ≤0,25 | 104 | 12 | 10 | Cnc | -18,3 |
| Control R6 | 147 | 0.78 | 1.01 | 155 | 3,7 | 0,36 | 102 | 13 | 17 | 21 | -17 |
| MVC R1 | 170 | 1.88 | 0,58 | 140 | 3,5 | 0,35 | 108 | 13 | 20 | Cnc | -10.5 |
| MVC R2 | 194 | 1,36 | 0,42 | 149 | 4,2 | 0,33 | 103 | 13.6 | 31 | Cnc | -11,4 |
| MVC R3 | 191 | 1,90 | 0,76 | 129 | 4,4 | 0,31 | 102 | 13.2 | 9,6 | Cnc | -12,5 |
| MVC R4 | 188 | 1,98 | 0,31 | 145 | 4,5 | 0.43 | 103 | 13 | 37 | 12 | -16 |
| MVC R5 | 165 | 0.99 | 0.95 | 141 | 3.8 | 0.26 | 101 | 12.1 | 21 | 9.5 | -13 |
| MVC R6 | 146 | 0.87 | 1.00 | 143 | 3.5 | 0.33 | 100 | 12.1 | 18 | 10 | -14 |
| MVC/ CDDP R1 | 293 | 3,01 | 1,99 | 198 | 6,7 | ≤0,25 | 134 | 13.2 | 21 | 6,3 | -18.7 |
| MVC/ CDDP R2 | 201 | 1,98 | 2,75 | 193 | 8,2 | ≤0,25 | 144 | 13,2 | 18 | 5,8 | -18.3 |
| MVC/ CDDP R3 | 199 | 3,2 | 3,33 | 189 | 5,8 | ≤0,25 | 137 | 13 | 18 | 6.1 | -17,3 |
| MVC/ CDDP R4 | 157 | 3,8 | 2,90 | 159 | 5,6 | 0,36 | 123 | 13 | 10 | 5,2 | -17 |
| CDDP R1 | 270 | 3,7 | 3,08 | 208 | 7,6 | 0,28 | 109 | 12 | 4 | Cnc | -10 |
| CDDP R2 | 310 | 3,8 | 2,64 | 211 | 6,8 | 0,28 | 150 | 15 | 1 | Cnc | -11 |
| CDDP R3 | 308 | 3,9 | 3,46 | 225 | 6,7 | 0,27 | 176 | 12 | 0.5 | Cnc | -12.0 |
| CDDP R4 | 298 | 4,1 | 2,97 | 212 | 6,4 | 0,32 | 184 | 12 | 5 | 5,5 | -12 |
| CDDP R5 | 293 | 3,7 | 1,97 | 207 | 5,8 | 0,33 | 193 | 14 | 8 | 5,4 | -13,2 |

**Hct:** total hematocrit **(**simian hematocrit: 35- 55%; murine hematocrit: 10-75%)

**BE(b) mmol/L** : <https://www.ncbi.nlm.nih.gov/pubmed/21755484>

**BEecf** excess extracellular fluid base.

**BE (B)** Current base excess is the amount of acid or base required to titrate 1 liter of blood to the normal pH of 7.40. It is useful to know how to compensate with doses of bicarbonate or ammonium chloride to correct metabolic imbalances (+3)

**THbc:** Total hemoglobin: 11.5 – 17.5 g/dL

**Supplementary Table 2.** Biochemical parameters obtained from BalbC NOD/SCID mice after treatments with MVC and/or CDDP.
